# Identification of a DRB3*011:01-restricted CD4^+^ T cell response against bovine respiratory syncytial virus fusion protein

**DOI:** 10.3389/fimmu.2023.1040075

**Published:** 2023-02-20

**Authors:** Bryan S. Kaplan, Amelia R. Hofstetter, Jodi L. McGill, John D. Lippolis, Junzo Norimine, Rohana P. Dassanayake, Randy E. Sacco

**Affiliations:** ^1^ Ruminant Diseases & Immunology Research Unit, National Animal Disease Center, Agricultural Research Service, United States Department of Agriculture, Ames, IA, United States; ^2^ Department of Veterinary Microbiology and Preventive Medicine, Iowa State University, Ames, IA, United States; ^3^ Department of Veterinary Medicine, University of Miyazaki, Miyazaki, Japan

**Keywords:** BRSV, F protein, BoLA class II, T cell, antigen presentation, cattle

## Abstract

Although Human Respiratory Syncytial Virus (HRSV) is a significant cause of severe respiratory disease with high morbidity and mortality in pediatric and elderly populations worldwide there is no licensed vaccine. Bovine Respiratory Syncytial Virus (BRSV) is a closely related orthopneumovirus with similar genome structure and high homology between structural and nonstructural proteins. Like HRSV in children, BRSV is highly prevalent in dairy and beef calves and known to be involved in the etiology of bovine respiratory disease, in addition to being considered an excellent model for HRSV. Commercial vaccines are currently available for BRSV, though improvements in efficacy are needed. The aims of this study were to identify CD4^+^ T cell epitopes present in the fusion glycoprotein of BRSV, an immunogenic surface glycoprotein that mediates membrane fusion and a major target of neutralizing antibodies. Overlapping peptides representing three regions of the BRSV F protein were used to stimulate autologous CD4^+^ T cells in ELISpot assays. T cell activation was observed only in cells from cattle with the DRB3*011:01 allele by peptides from AA249-296 of the BRSV F protein. Antigen presentation studies with C-terminal truncated peptides further defined the minimum peptide recognized by the DRB3*011:01 allele. Computationally predicted peptides presented by artificial antigen presenting cells further confirmed the amino acid sequence of a DRB3*011:01 restricted class II epitope on the BRSV F protein. These studies are the first to identify the minimum peptide length of a BoLA-DRB3 class II-restricted epitope in BRSV F protein.

## Introduction

Human respiratory syncytial virus (HRSV) is a significant cause of respiratory disease in infants worldwide that is estimated to infect nearly all children by two years of age (1, 2). It is estimated that worldwide, HRSV is responsible for the hospitalization of 3.2 million children (3) and the deaths of between 66,000-199,000 children under five years of age, many within the developing world (4). In addition, severe infection with HRSV within the first two years of life has been linked to the development of severe asthma (5, 6). Reinfection with HRSV has increasingly been recognized as a major cause of severe respiratory disease and hospitalizations in the elderly and those with underlying co-morbidities (7, 8). Currently, monoclonal antibodies are the sole treatment for respiratory disease caused by HRSV infection as there is no licensed vaccine and development of safe, effective vaccines against HRSV have been hindered in part due to the lack of an animal model capable of fully recapitulating the disease and immune response to infection (9).

HRSV, together with closely related bovine respiratory syncytial virus (BRSV), are also known as human orthopneumovirus and bovine orthopneumovirus, respectively, and combined with metapneumovirus comprise the family Pneumoviridae (10, 11). HRSV and BRSV have a single-stranded, negative sense RNA genome that is approximately 15.2 kb and encodes for 11 proteins (12, 13). BRSV is a significant respiratory pathogen of dairy and beef calves and a causative agent of bovine respiratory disease. The epidemiology of BRSV is similar to that of HRSV in that it causes outbreaks of high morbidity respiratory disease in young, naïve populations. BRSV is known to cause outbreaks affecting over 50% of animals in dairy and beef herds (14) with seropositive rates increasing with age up to 70% in adult cattle (15–17). The clinical course of disease for BRSV infected calves is very similar to that observed for HRSV infected humans with initiation of infection occurring in the upper respiratory epithelium and subsequent spread to the lower respiratory tract (18, 19). The immune response to BRSV in calves is characterized by the recruitment of T cells to the respiratory tract with M2, F, G, and N protein specific CD8^+^ T cells being notable (20–22) which is similar to the human T cell response to HRSV infection (23, 24). Additionally, BRSV infection of calves biases the cytokine response towards a Th2-type profile, similar to the response in humans, which is thought to predispose cattle to allergic disorders (25).

Antigen-specific CD4^+^ T cell responses are critical for immunity against viral and bacterial pathogens and a comprehensive understanding the dominant T cell epitopes is essential for rational vaccine design. The major histocompatibility complex class II (MHC class II) molecules, encoded by antigen presenting cells (APCs), bind to and present processed pathogen-derived peptides recognized by T cell receptor (TCR) molecules expressed on the surface of CD4^+^ T cells and recognition of peptide-MHC class II complexes by T cell receptors initiates multiple signaling cascades resulting in an antigen specific humoral immune response (26). The human genome encodes three highly polymorphic MHC class II gene loci, HLA-DR, -DP, and -DQ while the genome of cattle (*Bos taurus*) encodes two MHC class II proteins, bovine leukocyte antigen (BoLA) DR and DQ. The DR loci is comprised of one DRA gene and three DRB genes with DRB3 being the only functional gene while gene duplication has increased the number DQA and DQB alleles (27–29). To counter the paucity of functional MHC class II genes encoded in the bovine genome, the majority of allelic diversity in cattle is achieved through high rates of genetic polymorphism, particularly in the DRB3 gene (29, 30). Additionally, intra-haplotype and inter-haplotype pairing of the DQ molecules further increases the potential MHC class II repertoire of cattle (27, 28). The importance of diversity in the MHC class II loci of cattle is demonstrated by the differential CD4^+^ T cell responses to epitopes derived from common bovine pathogens, where specific DR or DQ alleles have been implicated in the mediation of a robust immune response (31–34).

The RSV F protein is a type I integral membrane glycoprotein responsible for mediating membrane fusion between viral and host cell membranes required for the initiation of infection (35, 36). Neutralizing antibody epitopes have been identified on the F protein trimer with antibody binding sites II, IV, and Θ being important targets for neutralizing antibodies (37–39). As the BRSV and HRSV F proteins are highly homologous, sharing 81% of their amino acid sequence (40), subunit vaccines incorporating the respective F proteins are currently in development for use in both cattle and humans (41–44). Helper T cell responses are critical for the development of antibody responses following vaccination or infection and few studies have identified allele-restricted CD4^+^ T cell epitopes on the F protein of BRSV and HRSV (45–48).

This aim of this study was to identify novel CD4^+^ T cell epitopes present on the F protein of the type strain BRSV-375. Peripheral blood mononuclear cells (PBMCs) were isolated from cows with a history of BRSV F protein exposure through vaccination. CD4^+^ T cell activation was assessed by interferon-γ (IFN-γ) production following the presentation of synthetic peptides by MHC class II defined antigen presenting cells. In experiments utilizing autologous and artificial APCs generated by transfecting human embryonic kidney 293 (HEK-293) cells with plasmids encoding BoLA DRA, DRB3, and CD80 molecules we identified a DRB3*011:01 restricted epitope and defined the minimum peptide length. An improved understanding of CD4^+^ T cell epitopes, and their allelic restriction, present on the BRSV F protein will enhance the understanding of the cellular response to RSV vaccines in cattle and humans.

## Materials and methods

### Cattle and determination of MHC alleles

Animal studies were performed in accordance with USDA National Animal Disease Center animal care and use protocols. Female Holstein Friesian cows were vaccinated annually beginning at 1 year of age with Vira Shield 6 (Elanco Animal Health, Indianapolis, IN, USA) which includes a BRSV component. Approximately 3mL of blood was collected from the jugular vein of cows into Vacutainer EDTA tubes (Beckton Dickinson, Franklin Lakes, NJ, USA). Genomic DNA was isolated using the QIAamp DNA Blood Mini kit (Qiagen, Valencia, CA, USA) according to manufacturer’s instructions. Genotyping of DRB3 alleles was performed *via* sequence-based typing (Sanger sequencing) utilizing previously described methods (49, 50) and restriction fragment length polymorphism (RFLP) as described by van Eijk et al. (30).

### Peptide libraries

Peptide design was informed by identifying homologous peptide sequences between the F proteins of HRSV and BRSV resulting in the identification of homologous BRSV peptide sequences for each previously described HRSV epitope (46, 51–54). The NetMHCpan (www.cbs.dtu.dk/services/NetMHCpan) server (55) and NetBoLAIIpan server (www.cbs.dtu.dk/services/NetBoLAIIpan) (56) were utilized to predict the binding of epitopes to specific class II BoLA alleles. Peptides predicted to bind BoLA class II alleles with high affinity (<500 nM) were selected. 11-mer peptides, with 10-mer overlaps (**Table 1**) and C-terminal truncated peptides (**Table 2**) were ordered from Mimotopes (Victoria, Australia). 15-mer peptides used in artificial antigen presenting cell (aAPC) studies were designed using the full length BRSV-375 protein and were synthesized by Vivitide (Gardner, MA). Peptides were diluted to a concentration of 2.5mM in dimethyl sulfoxide (Millipore Sigma, Burlington, MA, USA) and stored at -20°C until use.

**Table 1:** BRSV F protein overlapping peptide library sequences.

Set 1 (AA 84-118)		Set 2 (AA 249-296)		Set 3 (AA 32-65)	
Peptide #	Sequence	Peptide #	Sequence	Peptide #	Sequence
1	RYNNAVIE	28	TYMLTNSELLS	63	YQSTCSAVSRG
2	RYNNAVIEL	29	YMLTNSELLSL	64	QSTCSAVSRGY
3	RYNNAVIELQ	30	MLTNSELLSLI	65	STCSAVSRGYL
4	RYNNAVIELQS	31	LTNSELLSLIN	66	TCSAVSRGYLS
5	YNNAVIELQSL	32	TNSELLSLIND	67	CSAVSRGYLSA
6	NNAVIELQSLM	33	NSELLSLINDM	68	SAVSRGYLSAL
7	NAVIELQSLMQ	34	SELLSLINDMP	69	AVSRGYLSALR
8	AVIELQSLMQN	35	ELLSLINDMPI	70	VSRGYLSALRT
9	VIELQSLMQNE	36	LLSLINDMPIT	71	SRGYLSALRTG
10	IELQSLMQNEP	37	LSLINDMPITN	72	RGYLSALRTGW
11	ELQSLMQNEPA	38	SLINDMPITND	73	GYLSALRTGWY
12	LQSLMQNEPAS	39	LINDMPITNDQ	74	YLSALRTGWYT
13	QSLMQNEPASF	40	INDMPITNDQK	75	LSALRTGWYTS
14	SLMQNEPASFS	41	NDMPITNDQKK	76	SALRTGWYTSV
15	LMQNEPASFSR	42	DMPITNDQKKL	77	ALRTGWYTSVV
16	MQNEPASFSRA	43	MPITNDQKKLM	78	LRTGWYTSVVT
17	QNEPASFSRAK	44	PITNDQKKLMS	79	RTGWYTSVVTI
18	NEPASFSRAKR	45	ITNDQKKLMSS	80	TGWYTSVVTIE
19	EPASFSRAKRG	46	TNDQKKLMSSN	81	GWYTSVVTIEL
20	PASFSRAKRGI	47	NDQKKLMSSNV	82	WYTSVVTIELS
21	ASFSRAKRGIP	48	DQKKLMSSNVQ	83	YTSVVTIELSK
22	SFSRAKRGIPE	49	QKKLMSSNVQI	84	TSVVTIELSKI
23	FSRAKRGIPEL	50	KKLMSSNVQIV	85	SVVTIELSKIQ
24	SRAKRGIPELI	51	KLMSSNVQIVR	86	SVVTIELSKI
25	RAKRGIPELIH	52	LMSSNVQIVRQ	87	SVVTIELSK
26	AKRGIPELIHY	53	MSSNVQIVRQQ	88	SVVTIELS
27	KRGIPELIHYP	54	SSNVQIVRQQS		
		55	SNVQIVRQQSY		
		56	NVQIVRQQSYS		
		57	VQIVRQQSYSI		
		58	QIVRQQSYSIM		
		59	IVRQQSYSIMS		
		60	VRQQSYSIMSV		
		61	RQQSYSIMSVV		
		62	QQSYSIMSVVK		

**Table 2:** BRSV F protein C-terminal truncated peptide sequences.

Peptide	Sequence	Length
1	LINDMPITNDQK	12-mer
2	LINDMPITNDQ	11-mer
3	LINDMPITND	10-mer
4	LINDMPITN	9-mer
5	LINDMPIT	8-mer
6	INDMPITNDQK	11-mer
7	INDMPITNDQ	10-mer
8	INDMPITND	9-mer
9	INDMPITN	8-mer
10	INDMPIT	7-mer

### PBMC isolation and CD4^+^ T cell enrichment

Peripheral blood was collected from the jugular vein of adult Holstein-Friesian cows into 2x acid citrate dextrose buffer. PBMCs were isolated using SepMate-50 tubes preloaded with 15mLs Lymphoprep (Stem Cell Technologies, Vancouver, British Colombia, Canada) according to manufacturer’s instructions. Contaminating red blood cells were removed using a hypotonic lysis buffer. Cells were resuspended in complete RPMI (cRMPI) (ThermoFisher, Waltham, MA, USA) supplemented with 10% fetal bovine serum, nonessential amino acids, essential amino acids, sodium pyruvate, 2-mercaptoethanol, and penicillin/streptomycin. Monocytes were separated from lymphocytes by adherence in 100mm tissue culture dish at 37°C, 5% CO_2_ for 2 hours. Positive selection of CD4^+^ T cells was performed by magnetic bead isolation using anti-bovine CD4 mouse monoclonal antibody (IgG1 Clone CACT138A, Bio-Rad, Hercules, CA, USA) in conjunction with anti-mouse IgG microbeads and MACS LS columns (Miltenyi Biotec, Gaithersburg, MD, USA) according to manufacturer’s instructions. Enriched CD4^+^ T cell suspensions were resuspended at 4x10^6^ cells/mL in cRPMI. CD4^+^ T cell suspensions were found to be greater than 90% pure following flow cytometric analysis. Cell suspensions were labeled with the same mouse anti-bovine CD4 monoclonal antibody as above followed by goat anti-mouse IgG1-AlexaFluor 488 then analyzed on a FACS Symphony custom flow cytometer (BD Biosciences, Franklin Lakes, New Jersey, USA).

### Generation of Human Embryonic Kidney (HEK)-293 artificial antigen presenting cells (aAPCs)

Bovine DRA, DRB3, and CD80 genes were cloned into the mammalian expression plasmid pcDNA4/myc-His-C (ThermoFisher, Waltham, MA, USA) *via* Gibson assembly master mix (New England Biolabs, Ipswich, MA, USA) according to manufacturer’s instructions. RNA was isolated from PBMCs using Qiagen RNeasy Kit (Qiagen, Hilden, Germany) following manufacturer’s instruction. cDNA was produced using SuperScriptIII reverse transcriptase with oligo dT primer (ThermoFisher, Waltham, MA, USA). Inserts and plasmids were amplified using Q5 high-fidelity 2x Master Mix (New England Biolabs, Ipswich, MA, USA) with gene specific primers in **Supplemental Table 1** and used to transform TOP10 chemically competent E. coli (ThermoFisher, Waltham, MA, USA). Colonies were screened by Sanger sequencing with T7 promoter forward primer and BGH reverse primer. Positive colonies were cultured overnight in LB media, 100μg/mL carbenicillin and plasmids were isolated using ZymoPURE II plasmid midiprep kit (Zymo Research, Irvine, CA, USA).

To generate artificial antigen presenting cells, HEK-293 cells were transfected with BoLA-DRA (NCBI Gene ID 506214), BoLA-DRB3 (NCBI Gene ID 282530), and bovine CD80 (NCBI Gene ID 407131) encoding plasmids and selected with antibiotics. HEK-293 cells were obtained from the American Type Culture Collection (Manassas, VA, USA) and cultured in complete Eagle’s Minimum Essential Medium (EMEM) (ThermoFisher, Waltham, MA, USA) supplemented with antibiotic/antimycotic solution (ThermoFisher, Waltham, MA, USA) and 10% fetal bovine serum. On the day prior to transfection 1x10^6^ cells were seeded in each well of 6-well plates and cultured overnight at 37°C, 5% CO_2_. Just prior to transfection, the growth media was removed and replaced with 1mL fresh complete EMEM. Transfections were performed using Lipofectamine 2000 (ThermoFisher, Waltham, MA, USA) according to manufacturer’s instruction with 1μg of each plasmid. The day following transfection, the media was removed and replaced with complete EMEM containing 400μg/mL zeocin (ThermoFisher, Waltham, MA, USA). To obtain a homogenous population of co-transfected cells, single cell sorting was performed using a FACSAria Fusion cell sorter (BD Biosciences, Franklin Lakes, New Jersey, USA). Transfected cells were labeled with anti-BoLA DR-RPE, clone CC108 (Bio-Rad, Hercules, CA, USA) and anti-CD80-FITC, clone IL-A159 (Bio-Rad, Hercules, CA, USA). Double positive cells were sorted into 96-well plates and transferred to larger flasks once 90% confluence was achieved. To confirm transgene expression prior to use in antigen presentation assays, transfected HEK 293 cells were labeled as described above and analyzed on a FACS Symphony custom flow cytometer. Data was analyzed using Flow-Jo software (BD Biosciences, Franklin Lakes, New Jersey, USA).

### Enzyme-linked immunosorbent spot assay

Peptide-specific CD4^+^ T cells were quantified by assessing IFN-γ secretion in an ELISpot assay. The day prior to, 96-well multiscreen filter plates (MilliporeSigma, Burlington, MA, USA) were coated with mouse anti-bovine IFN-γ antibody (Bio-Rad, Hercules, CA, USA) at 4°C overnight. Two hours prior to the addition of cells, the plates were washed 6 times with PBS, 0.05% Tween-20 (PBST) and blocked with cRPMI at 37°C. Following the blocking step, plates were washed with PBST and 100μl cell suspension (1x10^5^ CD4^+^ cells/well, 2x10^4^ APC/well) was added to wells. Cells were stimulated in 100μl of solution containing peptide (final concentration 20μM) or PHA (final concentration 5μg/mL) diluted in cRPMI. For blocking experiments anti-bovine MHC class II DR monoclonal antibody clone TH14B, anti-bovine MHC class II DQ monoclonal antibody clone TH81B were preincubated with adherent cells (2-hours) or aAPCs (20min) prior to incubation with purified CD4^+^ T cells. Anti-bovine IL-4 monoclonal antibody clone CC303 (Bio-Rad, Hercules, CA, USA) or IgG2 monoclonal antibody clone COLIS205c (Washington State University monoclonal antibody center, Pullman, WA, USA) were used as isotype control. Antibodies were diluted to concentrations of 16 and 160μg/mL prior to incubation with APCs; final concentrations of anti-DR and anti-DQ antibodies were 0.8μg/mL and 8μg/mL and those of control antibodies were 0.8μg/mL. Plates were incubated for 20 hours at 37°C, 5% CO_2_. Following incubation plates were washed with PBST then incubated with mouse anti-bovine IFN-γ clone CC302 (Bio-Rad, Hercules, CA, USA) at 5μg/mL in PBS, 1% BSA for 2 hours at 37°C. Plates were then washed with PBST and stained using 100μl Vector ABC AP standard (Vector Laboratories, Burlingame, CA, USA) for 45 minutes at room temperature followed by 50μl Vector Blue AP substrate III for 30 minutes at room temperature. Plates were washed 2x with 300μl distilled water and allowed to dry overnight prior to visualization with an ImmunoSpot Analyzer (Cellular Technology Limited, Cleveland, OH, USA).

### Statistical analysis

The mean values for each group were analyzed using one-way ANOVA test with multiple comparisons or the Mann-Whitney nonparametric test. Prior to this, normality tests (D’Agostino & Pearson, Anderson-Darling, Shapiro-Wilk, and Kolmogorov-Smirnov) were performed and the data are not normally distributed. Differences were considered significant when P value < 0.05. Data were analyzed with GraphPad Prism 9 (GraphPad Software, La Jolla, CA, USA).

## Results

### Genotype and vaccination history of Dairy Cows

To determine the presence of specific BoLA-DRB3 alleles in the USDA-ARS NADC dairy herd, Sanger sequencing and RFLP analysis were performed on genomic DNA extracted from whole blood. A total of 18 Holstein-Friesian cows were screened and 10 different DRB3 alleles were identified (**Table 3**) that had been previously reported in the Immunopolymorphism Database (57). The allele detected with the highest frequency was *011:01 (n=7 animals) followed by *015:01 (n=6 animals) and *014:01:01 (n=4 animals). All animals received multiple doses of a commercial BRSV vaccine. The number of doses received varied with ~78% (14/18) receiving at least 3 doses.

**Table 3:** BoLA-DRB3 alleles of dairy cows screened for responsiveness to BRSV F protein peptides.

Animal^1^	RFLP		Allele		Epitope Specificity	Vaccination history, n^2^
968	24	11	*01:01	*009:02	N	8
**1141**	22	16	*011:01	*015:01	Y	10
1142	24	11	*027:03	*009:02	N	11
1143	16	16	*015:01	*015:01	N	11
1146	16	16	*026:01	*015:01	N	10
1147	8	8	*012:01	*012:01	N	10
**1310**	21	22	*008:01	*011:01	Y	4
**1503**	16	22	*015:01	*011:01	Y	3
1504	16	16	*015:01	*015:01	N	4
**1613**	27	22	*014:01:01	*011:01	N	1
*1930*	22	22	*011:01	*011:01	Y	3
*1944*	27	16	*014:01:01	*014:01:01	N	2
*1947*	24	24	*001:01	*001:01	N	2
2927	27	7	*014:01:01	*002:01	N	6
**2938**	22	16	*011:01	*015:01	Y	5
2939	27	27	*014:01:01	*014:01:01	N	7
**2942**	21	22	*008:01	*011:01	Y	5
*8470*	8	8	*012:01	*012:01	N	2

^1^Animal numbers listed in bold used in C-terminal truncated peptide studies, bold and underlined in overlapping peptide library studies, and italics in artificial APC studies.

^2^Number of standard vaccinations administered at time of study.

### DRB3*011:01 presents synthetic peptides spanning BRSV F protein region AA 249-296

**Figure 1:**
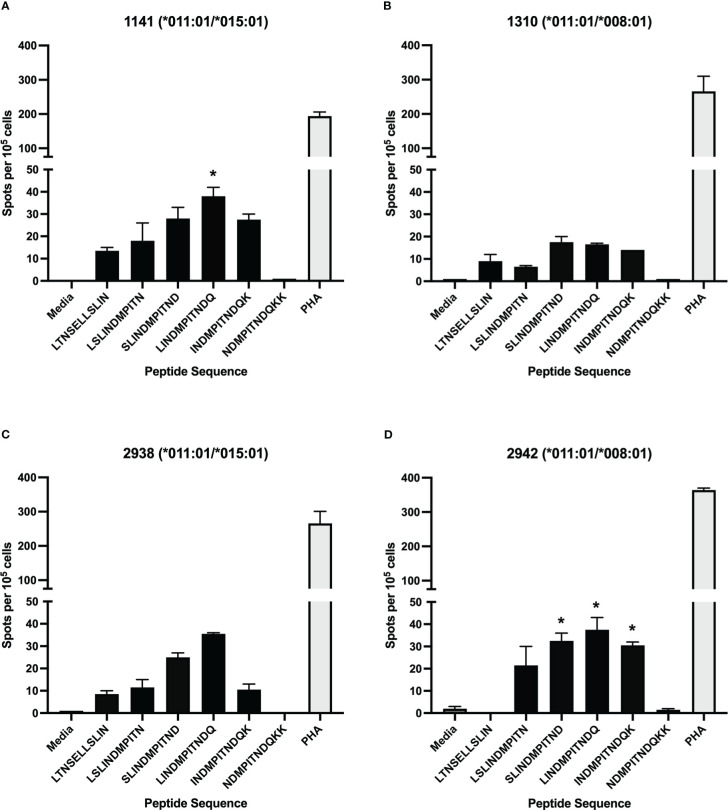
Stimulation of CD4^+^ T cells with synthetic, overlapping peptides from the BRSV-375 F protein. CD4^+^ T cells from dairy cows were positively selected and cultured with autologous antigen presenting cells loaded with synthetic peptide. T cell activation was assessed *via* IFN-γ secretion using ELISpot assays. Only cells from DRB3*011:01 positive animals responded to stimulation with five out of 88 peptides tested. T cell responses from animals 1141 **(A)**, 1310 **(B)**, 2938 **(C)**, and 2942 **(D)** displayed a measurable T cell response. Peptides sharing N-terminal amino acid with BRSV-375 F protein residue 253 generated a slight response while those with N-terminal amino acids matching residues 257-260 displayed a larger response. T cells were stimulated with PHA at a concentration of 5μg/mL as a positive control, and cRPMI media as a negative control. Data presented as mean ± SEM, p < 0.05 was considered significant and indicated by asterisk.

To assess the position of potential T cell epitopes on the BRSV F protein, a library of overlapping peptides, with 10-mer overlaps, was designed focusing on three regions of the full-length protein shown to have high homology to regions of HRSV F protein that harbor T cell epitopes. The three sets of peptides, 88 peptides in total ranging in length from 8-11 amino acids, corresponded to three regions (84-118, 249-296, and 32-65) of the BRSV F protein amino acid sequence (**Table 1**). Peptide-specific CD4^+^ T cells responses were assessed *via* IFN-γ production in ELISpot assays. Only CD4^+^ T cells isolated from cows harboring the DRB3*011:01 allele (1141, 1310, 2938, and 2942) responded to stimulation with BRSV F protein derived peptides while no response was observed in cells from cows lacking the DRB3*011:01 allele (data not shown). Interestingly, only peptides derived from the BRSV F protein region spanning AA 249-296 produced a measurable response. In total, 5 out of 35 peptides from set 2 were capable of inducing IFN-γ from cells isolated from DRB3*011:01 cattle (**Figure 1**). The most robust T cell responses were observed following stimulation with peptides 38 (SLINDMPITND) and 39 (LINDMPITNDQ) with slightly reduced levels of T cell responses observed against peptides 37 (LSLINDMPITN) and 40 (INDMPITNDQK). In contrast, a dimished response to peptide 31 (LTNSELLSLIN), compared to peptides 37 and 40, was observed while stimulation with peptide 41 (NDMPITNDQKK) failed to elicit a detectable response. The most robust response was observed for CD4^+^ T cells collected from cows 1141 and 2942 followed by 2938; while cow 1310 displayed a similar response pattern, the intensity of the response was consistently lower compared to the other three. Together, these results identify a novel CD4^+^ T cell epitope in the BRSV F protein that is restricted to DRB3*011:01 and begin to define the core epitope sequence.

Interestingly, the magnitude of the T cell response correlated with the vaccination history of each cow (**Table 3**). The most robust T cell responses were observed in DRB3*011:01 positive cows that received more than 3 annual vaccines (1141, 1310, 2938, and 2942). Further, the responses were graded with the highest T cell responses being observed from cow 1141 that had received 10 doses of annual vaccine and cow 2942, that received 5 doses. T cell responses from cows 1310 and 2938, that received 4 and 5 doses, respectively, were less diminished compared those observed for cows 1141 and 2942. In contrast, cows that received 3 or fewer annual vaccine doses T cell responses greatly reduced (1503) or undetectable (1613). These data suggest repeat immunization correlates to increased BRSV specific CD4^+^ T cell responses and the existence of a threshold number of doses required for detectable T cell levels.

### DRB3*011:01 presents peptides with the core sequence INDMPITND in BRSV F protein T cell epitopes

**Figure 2:**
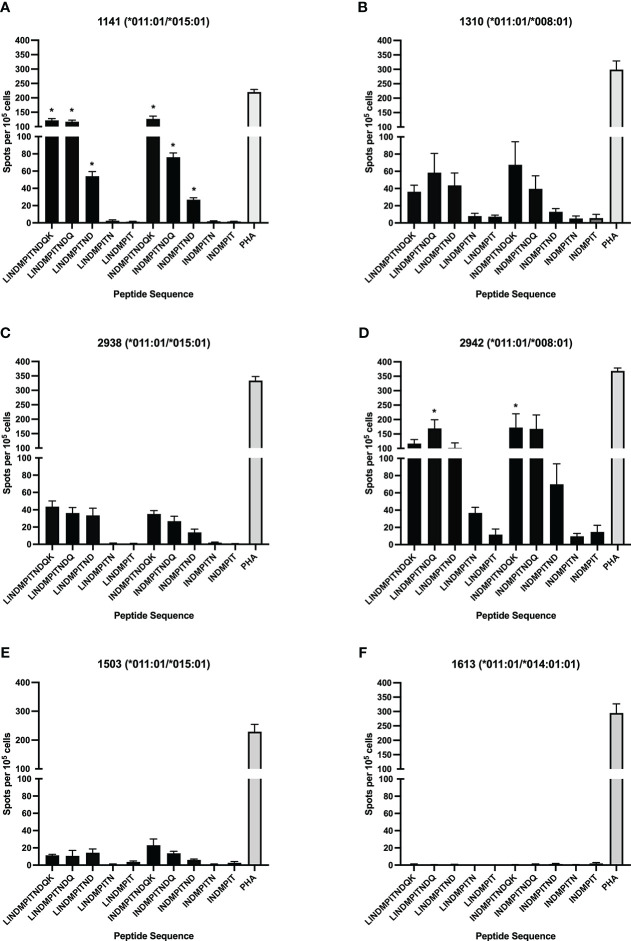
Identification of the minimum peptide of a CD4^+^ T cell epitope in BRSV-375 F protein. C-terminal truncated peptides were used to stimulate CD4^+^ T cells in culture with autologous DRB3*011:01 antigen presenting cells. T cell stimulation was observed for peptides greater than 8 amino acids in length. The 9-mer minimum peptide sequence of INDMPITND was identified. T cells were stimulated with PHA at a concentration of 5μg/mL as a positive control, and cRPMI media as a negative control. Cows 1141 **(A)** and 2942 **(D)** had a significantly higher response to peptides containing the core INDMPITND sequence while T cells from 1310 **(B)**, 2938 **(C)**, 1503 **(E)** responded to these peptides, the results were not significant. T cells collected from cow 1613 **(F)** did not produce an observable response to peptide stimulation. Data presented as mean ± SEM, p < 0.05 was considered significant and indicated by asterisk.

To further define the DRB3*011:01-restricted epitope core sequence and minimum length a library of C-terminal truncated peptides centered on the region of the BRSV F protein, LINDMPITNDQK, was used in antigen presentation assays. The truncated peptide library is composed of peptides ranging in length from 7-mer to 12-mer (**Table 2**). To assess allelic restriction and the effects of peptide length, CD4^+^ T cells from six DRB3*011:01 positive cattle 1141, 1310, 1503, 2938, 2942 (**Figure 2**) were stimulated with individual peptides from the truncated peptide library. The most robust T cell responses were observed for peptides ranging in length from 10-12 residues. Similar levels of T cell response were observed following stimulation with a N-terminal leucine or isoleucine (peptides 1-3 and 6-8). Removal of amino acid residues from the C-terminus of each peptide core resulted in a sequence dependent reduction of T cell stimulation. Removal of the C-terminal aspartate residue from peptides 3 and 8 significantly reduced the induction of IFN-γ as observed following stimulation with peptides 4 and 9. As observed previously, animals 1141 and 2942 displayed the greatest response out of all DRB3*011:01 cows tested, while no response was observed from 1613, likely due to this cow receiving only a single vaccine dose.

**Figure 3:**
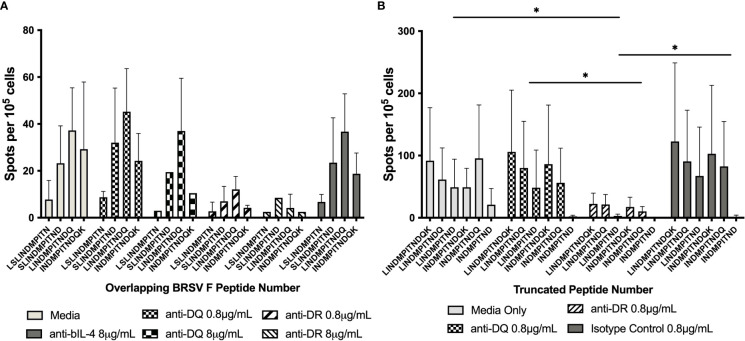
Inhibition of BoLA DR, but not BoLA DQ, significantly reduces CD4^+^ T cell activation. Antigen presenting cells were incubated with antibodies against BoLA-DR or BoLA-DQ molecules prior to stimulation with synthetic overlapping peptides **(A)** or C-terminal truncated peptides **(B)**. Antibodies raised against MHC class II molecules were provided as mouse ascites fluid. A reduction of in T cell stimulation was observed only with following pre-treatment with anti-BoLA DR antibodies **(A)**. Antibodies were purified to ensure the reduction in T cell stimulation was due to antibody binding and not background from ascites fluid **(B)**. Anti-bovine IL-4 antibody was used as an isotype control. Cells were cultured in cRPMI as a media only control. Data is presented as mean ± SEM, p < 0.05 was considered significant and indicated by asterisk.

To ensure the response to peptide stimulation was mediated through MHC class II, blocking experiments where autologous APCs were incubated with antibody against either BoLA DR or DQ molecules prior to incubation with CD4^+^ T cells and stimulation with peptide were performed (**Figure 3A**). Antigen presenting cells from animals 1142, 1310, 2938, and 2942 displayed similar levels of stimulation following pretreatment with media, the anti-BoLA-DQ antibody, TH81A, or an isotype control antibody. In contrast, a significant reduction in T cell stimulation was observed when APCs were incubated with the anti-DR antibody, TH14B, prior to treatment with peptide. As the initial blocking experiments utilized ascites fluid, antibodies were purified with protein G and the purified antibody was used in subsequent blocking experiments (**Figure 3B**). No reduction of T cell stimulation was observed when APCs were incubated with purified anti-DQ antibody compared to media only and isotype controls. Conversely, pre-treatment of APCs with purified anti-DR antibody significantly reduced the stimulation of CD4^+^ T cells. Together these results show the DR MHC class II molecule is responsible for mediating presentation of peptide to BRSV F protein to specific CD4^+^ T cells.

### DRB3*011:01 artificial APCs Present F protein peptides with INDMPITND core sequence

**Figure 4:**
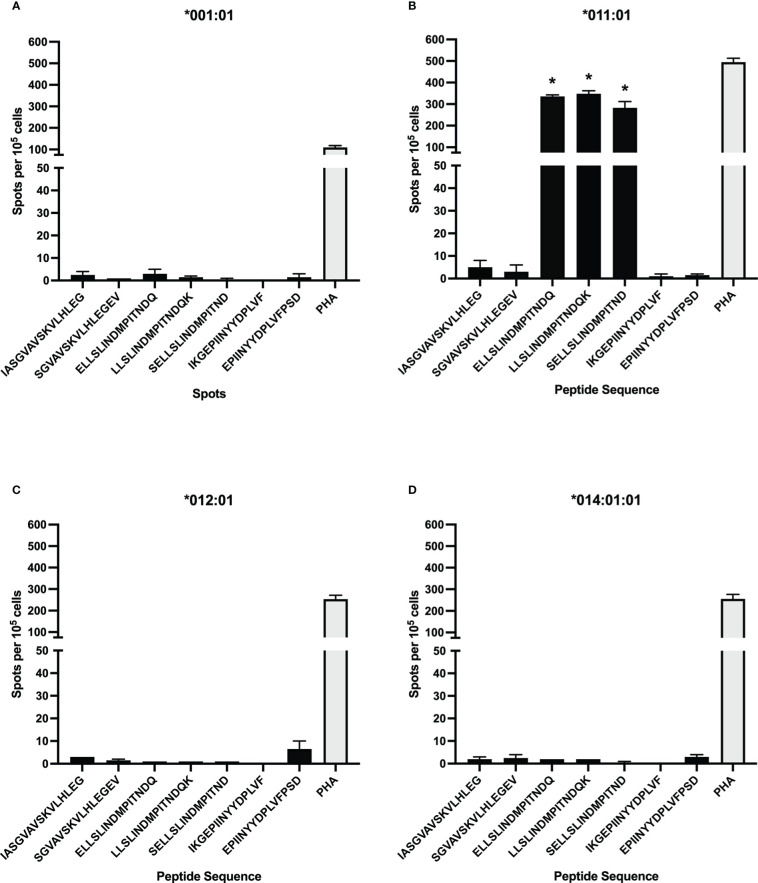
CD4^+^ T cell response to computationally predicted peptides presented by artificial antigen presenting cells (aAPC). Synthetic peptides computationally predicted to bind strongly to select BoLA-DRB3 alleles used to stimulate CD4^+^ T cells after loading to aAPCs expressing BoLA-DR and CD80 molecules. DRB3*011:01 predicted peptides, with a core sequence of INDMPITND, were observed to induce T cell activation **(B)** while those predicted to bind to *001:01 **(A)**, *012:01 **(C)**, and *014:01:01 **(D)** did not. Cells were stimulated with 5μg/mL PHA as a positive control. Data presented as mean ± SEM, p < 0.05 was considered significant and indicated by asterisk.

**Figure 5:**
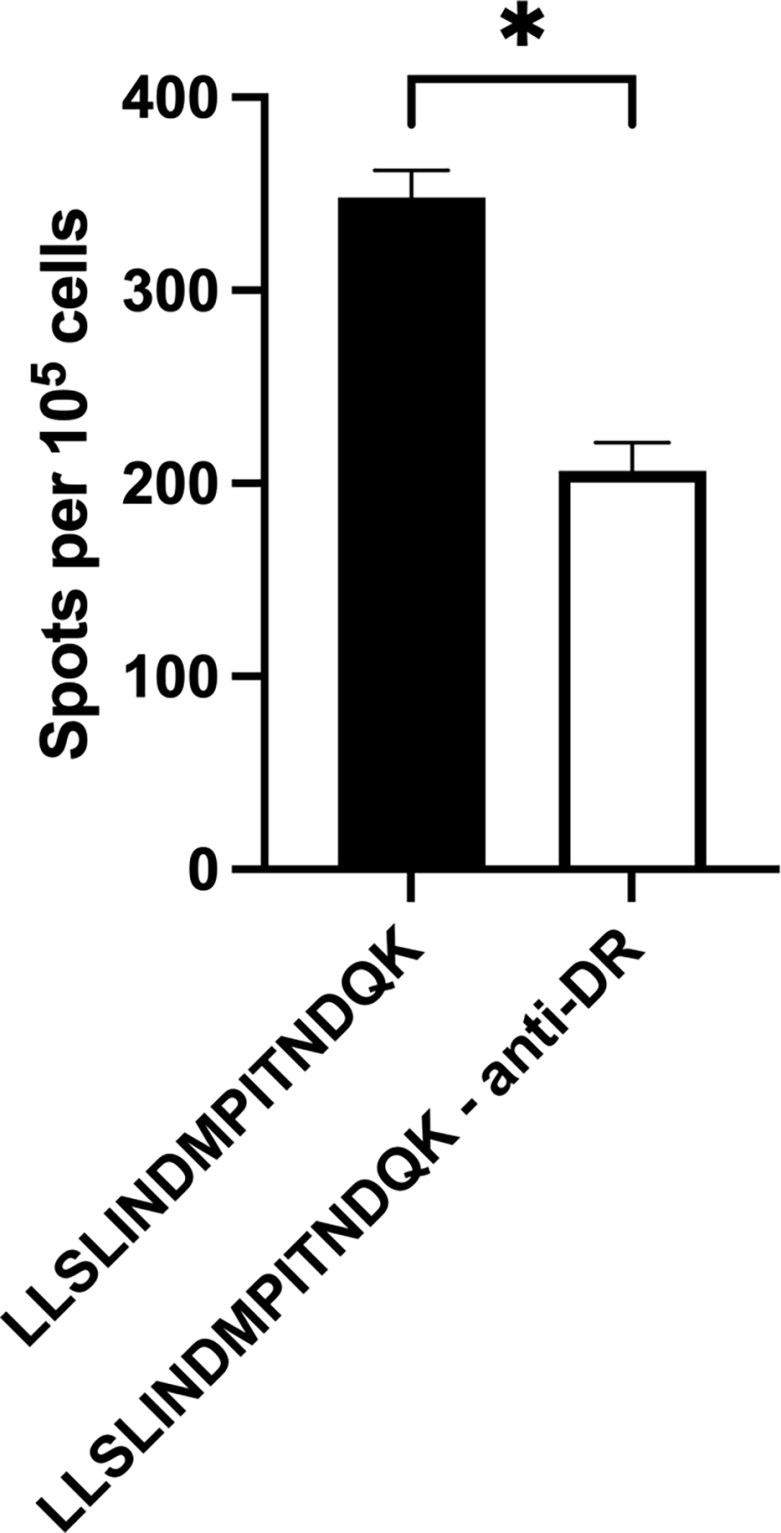
Inhibition of BoLA DR significantly reduces T cell activation. Artificial antigen presenting cells incubated with anti-BoLA DR antibody significantly reduced the number of T cells responding to BRSV-375 F protein peptide. Peptide 4 sequence: LLSLINDMPITNDQK. Data presented as mean ± SEM. Statistical significance was determined using the Mann-Whitney nonparametric test, P < 0.05 indicated by asterisk.

To test additional BoLA-DR alleles for BRSV F protein peptide presentation, aAPCs were generated by transfecting HEK-293 cells with plasmids encoding four different BoLA-DR molecules common to the NADC dairy herd and bovine CD80. Following single cell sorting and clonal expansion, HEK-293 cells co-expressing each BoLA-DRB3 allele, *001:01, *011:01, *012:01, and *014:01:01, and CD80 were assessed by flow cytometry. For each aAPC line, the proportion of DR and CD80 co-expressing cells was greater than 90% (**Supplemental Figure 1**). A library of 15-mer peptides computationally predicted to bind each cloned BoLA-DR allele was synthesized and used in antigen presentation studies (**Table 4**). At least two peptides predicted to react to each cloned allele was synthesized with each centered around a 9-mer core sequence. The core sequence (INDMPITND) for peptides 3, 4, 5 was shown to be critical for antigen presentation by DRB3*011:01 molecules as previously shown in **Figure 2**. Artificial APCs were loaded with each of the seven peptides and assessed for their ability to stimulate BoLA-DR matched CD4^+^ T cells (**Figure 4**). Only peptides predicted to bind DRB3*011:01 were observed to induce a response when cultured with autologous T cells (**Figure 4B**). To ensure class II molecules were responsible for mediating T cell activation, we pre-treated artificial APCs with the anti-DR antibody TH14B prior to peptide loading. As shown in **Figure 5**, a significant reduction in T cell stimulation was observed in cells pre-treated with antibody compared to untreated cells. These results confirm the presence of a BRSV F protein CD4^+^ T cell epitope that includes the core sequence INDMPINTND. Further, this epitope is restricted to presentation by the DRB3*011:01 allele.

**Table 4:** Computationally predicted BoLA Class II peptides.

Peptide	Sequence	Core Sequence	Predicted Allele^1^
1	IASGVAVSKVLHLEG	VAVSKVLHL	*01:01, *014:01:01
2	SGVAVSKVLHLEGEV	VAVSKVLHL	*01:01, *014:01:01
3	ELLSLINDMPITNDQ	INDMPITND	*011:01
4	LLSLINDMPITNDQK	INDMPITND	*011:01
5	SELLSLINDMPITND	INDMPITND	*011:01
6	IKGEPIINYYDPLVF	IINYYDPLV	*012:01
7	EPIINYYDPLVFPSD	IINYYDPLV	*012:01

^1^Predicted to bind strongly to listed allele by NetBoLAIIPan-1.0 software.

## Discussion

The design and improvement of novel vaccine platforms and strategies against pathogens to which traditional, inactivated vaccines failed to win licensure requires comprehensive functional knowledge of the immune response against a specific pathogen and vaccine antigen. Vaccines against respiratory viruses are developed with the aim to induce neutralizing antibodies thereby preventing infection and resulting in rapid clearance of virus particles. In the 1960’s, clinical trials using a formalin-inactivated whole-virion HRSV vaccine failed following vaccine-enhanced disease in children (58). The observed enhanced disease is now known to have been mediated by immune complex deposition and complement activation resulting from the production of poorly neutralizing antibodies (59–61) and the induction of a Th2 immune response (62). Recent RSV vaccine development efforts focus on the F protein as it is a major target for neutralizing antibodies with results from a phase 1 clinical trial using a prefusion conformation stabilized F protein, DS-Cav1, showing increases in neutralizing antibody titers in human volunteers (63). Neutralizing antibody titers, induced following vaccination or natural infection, contract over time and thus measuring the magnitude and longevity of T cell responses to pathogens is critical to gauge vaccine immunogenicity and longevity of induced response.

This study is the first to map CD4^+^ T cell epitopes in the F protein of a North American BRSV isolate, BRSV 375. For these studies, the number of IFN-γ secreting CD4^+^ T cells was quantified following stimulation with autologous or artificial antigen presenting cells loaded with synthetic peptides in an ELISpot assay. PBMCs from cattle determined to be positive for several BoLA-DRB3 alleles previously shown to respond to specific epitopes in other diseases of cattle, namely the DRB3*011:01, *012:01, *015:01 alleles (**Table 3**) (33, 64). Preliminary studies found only cells from DRB3*011:01 cattle responded to stimulation with the overlapping peptide library (data not shown). These results are consistent with previous epitope mapping studies where the DRB3*011:01 allele has been shown to readily present peptides from a diverse group of bovine pathogens including foot-and-mouth disease virus (65) and the blood parasite *Anaplasma marginale* (28, 33, 64). Recently, the BoLA class II DRB3*009:02 allele has been shown to be significantly associated with undetectable levels of bovine leukemia virus proviral loads in cattle suggesting antigen presentation through this allele is central in the development of an effective immune response against bovine leukemia virus (34, 66), further highlighting the importance of specific BoLA class II alleles in protection from viral infection. As the DRB3*011:01 allele is capable of presenting peptides from a diverse group of pathogens, it would be of interest to study the antibody profiles and clinical outcomes of these cattle following BRSV infection. Further, studies addressing the T cell receptor repertoire may shed light on epitope usage as this is likely to be influenced by MHC class II haplotype.

In this study, a previously unknown CD4^+^ T cell epitope on the BRSV F protein was identified. The epitope has a core sequence of INDMPITND and spans amino acids 261 to 269. This sequence is conserved between the North American type strain BRSV 375 and the European type strain BSRV Snook (data not shown) as well as present within HRSV A and B subtype F proteins (67). Interestingly, this epitope resides in a stretch of exposed surface shared by antibody binding sites II and III, whereas the therapeutic monoclonal palivizumab binds to antigenic site II (68). The region of F protein spanning residues 249-296 has previously been shown to contain other epitopes recognized by human (48) and bovine (45) CD4^+^ T cells though T cell epitopes are present outside of this region (47). The results presented herein show CD4^+^ T cells reacted with autologous APC presented 11-mer peptides with N-terminal amino acid at residues 252, 258, 259, 260, and 261 (**Figure 1** and **Table 1**). Further characterization of this region using c-terminal truncated peptides identified the minimum peptide required for CD4^+^ T cell stimulation (**Figure 2** and **Table 2**). Fogg et al. (45) have previously identified multiple epitopes outside of the 249-296 region presented by other DRB3 alleles. In an attempt to identify other allele specific epitopes, a library of peptides computationally predicted to bind additional DRB3 alleles *001:01, *012:01, and *014:01:01 (**Figure 4** and **Table 4**) was used in antigen presentation studies. In ELISpot assays, purified CD4^+^ T cells cultured with artificial APCs did not produce IFN-γ in response to antigen presentation. It is possible additional DRB3-restricted epitopes are present in the BRSV 375 F protein and further studies with expanded peptide libraries spanning the entirety of the F protein will be critical for the identification of additional CD4^+^ T cell epitopes.

Genetic diversity of the MHC class II loci is essential for the presentation of peptides derived from the staggering diversity of pathogens encountered by individuals of a species. The human genome encodes genes for three MHC class II proteins (HLA-DR, -DP, and -DQ) while the bovine genome encodes for only two (BoLA-DR and -DQ) with polymorphisms in both species increasing genetic diversity (26, 29). Antigenic peptide presentation can be restricted to one isotype of the MHC class II molecules, i.e. BoLA-DR, or presented by a combination of MHC class II proteins, termed interhaplotype pairing. To determine if this epitope is restricted to presentation through BoLA-DR, we performed blocking experiments where APCs were incubated with antibodies against either BoLA-DR or BoLA-DQ molecules prior to co-culture with CD4^+^ T cells. Inhibition of T cell stimulation was observed following APC treatment with anti-DR antibodies (**Figures 3**, **5**), indicating the epitope is restricted to DRB3*011:01. Despite lack of response for other alleles tested, peptides maybe presented by DQ alleles, which were not examined. Studies assessing antigenic peptides derived from *Anaplasma marginale* have shown cattle utilize either DR or DQ molecules or present peptides *via* interhaplotype pairing (28, 33). The studies presented above did not investigate the potential for antigen presentation with DQ or interhaplotype pairing. Additional studies would be useful in further characterizing the role of BoLA-DQ following vaccination or infection with BRSV.

Development of novel vaccines to existing and emerging pathogens is a high priority and the development of animal models that fully recapitulate disease and the ensuing immune response are invaluable. As BRSV is closely related to HRSV and causes similar disease, the neonatal calf model is considered to be invaluable for the development novel vaccines against HRSV (69). Recently a polyanhydride nanovaccine incorporating the F protein of BRSV (41, 70) was shown to be effective in preventing severe disease in calves. Polyanhydride nanovaccines were highly immunogenic, inducing strong antibody and cellular responses to both BRSV surface glycoproteins and reduced virus titer and lung pathology following challenge. Additionally, a study assessing a prefusion-stabilized BRSV F protein vaccine was also shown to be immunogenic and protect calves from disease following challenge (44). These studies underscore the importance of the F protein in BRSV immunity. It is of interest to assess the antigen specific T cell response following vaccination including determination of the magnitude and longevity of the CD4^+^ T cell response. These data will be useful in the refinement of vaccine formulations and dosing schedules. The calf model of BRSV infections is ideally suited to address these questions as the amino acid sequence of the BRSV and HRSV F sequences are highly homologous and share common T cell epitopes and antibody binding sites. The data presented here builds on previous studies by expanding our knowledge of CD4^+^ T cell epitopes present in the BSRV F protein and will aid in defining the immune response to BRSV infection as well as the development of vaccines for cattle and humans.

## Data availability statement

The original contributions presented in the study are included in the article/**Supplementary Material**. Further inquiries can be directed to the corresponding author.

## Ethics statement

The animal study was reviewed and approved by National Animal Disease Center/USDA/ARS IACUC.

## Author contributions

The experiments were conceived by BK, AH, JM, RD, and RS and were performed by BS and AH. Analyses were conducted by BK, AH, JN. The manuscript was written by BK and RS and edited by BK, AH, JM, JN, RD, and RS. All authors contributed to the article and approved the submitted version.

## Supplementary material

Supplementary Figure 1 Artificial antigen presenting cells co-expressing BoLA DR and CD80 molecules. HEK-293 cells were transfected with plasmids encoding BoLA DR and CD80. Single cell populations were isolated, expanded, and assessed for transgene expression *via* flow cytometry. Gating strategy for phenotyping analysis (A). Population of co-transfected HEK 293 cells with each BoLA DR allele (B).

Supplementary Table 1 Primer sequences used to amplify DRA, DRB3, and CD80 for aAPC generation.
