# Supplementary figures and images for: Identification of a DRB3*011:01-restricted CD4+ T cell response against bovine respiratory syncytial virus fusion protein
Source: Front Immunol. 2023 Feb 20;14:1040075. doi: 10.3389/fimmu.2023.1040075 (PMC9986546; doi:10.3389/fimmu.2023.1040075)

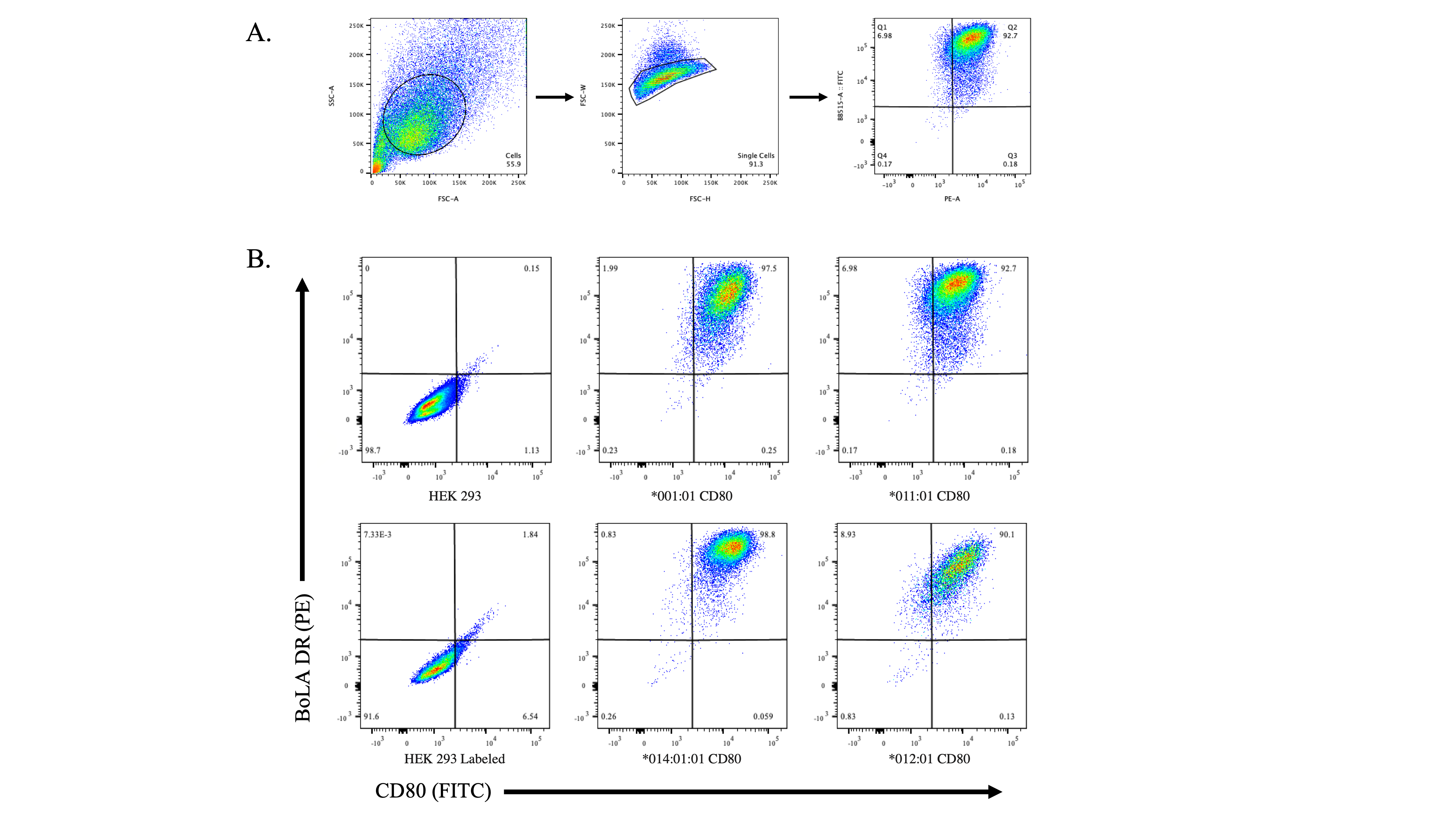

Supplement: Supplementary Figure 1 — Artificial antigen presenting cells co-expressing BoLA DR and CD80 molecules. HEK-293 cells were transfected with plasmids encoding BoLA DR and CD80. Single cell populations were isolated, expanded, and assessed for transgene expression via flow cytometry. Gating strategy for phenotyping analysis (A). Population of co-transfected HEK 293 cells with each BoLA DR allele (B). [file Image_1.tiff]
